# Suicide risk among adolescents and young adults after cancer diagnosis: analysis of 34 cancer groups from 2009 to 2019

**DOI:** 10.1007/s11764-023-01358-5

**Published:** 2023-03-17

**Authors:** Irmina Maria Michalek, Florentino Luciano Caetano dos Santos, Urszula Wojciechowska, Joanna Didkowska

**Affiliations:** 1 Polish National Cancer Registry, Maria Sklodowska-Curie National Research Institute of Oncology, Warsaw, Poland; 2 Epidemiology Department, Maria Sklodowska-Curie National Research Institute of Oncology, Warsaw, Poland

**Keywords:** Cancer, Suicide, Risk, SMR, Adolescents, Young adults

## Abstract

**Purpose:**

We aimed to identify granular groups with an increased risk of suicide among adolescents and young adult (AYA) patients with a previous malignant neoplasm diagnosis.

**Methods:**

We deployed a cohort of all cases of primary malignant neoplasms diagnosed between the 1st of January 2009 and the 31st of December 2019 among individuals aged 15–39 years registered in the Polish National Cancer Registry. To assess the risk of suicide in comparison with the general AYA population, we calculated sex–age–year standardized mortality ratios (SMR) with 95% confidence intervals (CI).

**Results:**

A total of 50,298 cancer patients (22,111 men and 28,187 women) were included in this study. The risk of suicide for AYA after cancer diagnosis was 2.39-fold higher than that for AYA in the general population (SMR 2.39, 95% CI 1.69 to 3.28). The risk in women (SMR 4.18, 95% CI 1.68 to 8.62) was higher than that in men (SMR 2.18, 95% CI 1.48 to 3.09). A significantly higher risk of suicide was observed in men with testicular cancer (SMR 2.46, 95% CI 1.37 to 4.05).

**Conclusions:**

Polish AYA diagnosed with cancer had an almost 2.5-fold higher risk of suicide than the general AYA population. The particular risk group was men with testicular cancer within 2–3 and 5–10 years after cancer diagnosis.

**Implications for Cancer Survivors:**

To better identify patients at risk of suicide, there is a need to create or adapt screening tools, educate cancer care providers and family physicians, and integrate psychological services into select cancer care specialties.

**Supplementary Information:**

The online version contains supplementary material available at 10.1007/s11764-023-01358-5.

## Introduction

On a global scale, adolescents and young adults (AYA), that is, people aged 15–39 years, account for approximately 7% of all incident cancer cases annually (1,233,225 out of 18,094,716 cancer cases in 2020) [1].

There is a clear link between instabilities in the developmental trajectory of the AYA and a higher likelihood of depression [2–4]. Regardless of the time of cancer diagnosis (during higher education, early career, or starting a family), glitches in anticipated life responsibilities and roles are a considerable psychological burden.

A comprehensive review of the literature shows that AYA diagnosed with malignant neoplasms are at a higher risk of suicide than those in the general population [5]. Although the risk is lower when compared to older cancer patients, there is still a need to define in detail the groups at an increased risk of suicide among AYA. An in-depth understanding of these issues is particularly important given the AYA relatively better survival rates than the overall cancer patient population (US 5-year survival rate 85.5% vs. 68.1%) [6, 7].

While the literature on suicide in the general cancer patient population is extensive [8], little is known about particular risk groups within the AYA population. In addition, although the two most prominent studies on this topic were based on the same SEER database, with negligible differences in the range of periods covered and the number of cases included [9, 10], their results were somewhat inconsistent, and calculation methods seem to influence the analysis results significantly. And so, according to Heynemann et al., the risk of suicide among American AYA with cancer was 34.1-fold higher than that of the general population [9], and, according to Yang et al., it was approximately 1.23 times higher [10]. Therefore, the generalizability of published research on suicide risk in AYA cancer patients is problematic.

Our study aimed to identify groups with an increased risk of suicide among AYA patients with a previous cancer diagnosis.

## Materials and methods

This sub-study was sourced from the Polish Suicidality in Cancer Patients (PolSCa) study population. PolSCa is a cohort study based on data from the Polish National Cancer Registry (PLCR) that covers all Polish cancer cases. The included cases encompassed all primary malignant neoplasms, except non-melanoma skin cancers (C00-C43, C45-C76, and C80-C96, according to ICD-10) diagnosed between the 1st of January 2009 and the 31st of December 2019. In this sub-study, we followed the AYA definition accepted by the American National Cancer Institute and Global Burden of Disease study and included all individuals who were 15–39 years old on the day of cancer diagnosis. The analysis of patients with two or more independent coexisting malignant neoplasms included only the most recent diagnosis of primary malignant neoplasm. The PLCR avoids multiple registrations of the same patient using a unique Polish personal identification number (PESEL). Details of the PLCR operation have been thoroughly described elsewhere [11]. The endpoints of follow-up encompassed suicide, death due to other causes, or the 31st of December 2019, whichever occurred first. Data on deaths due to suicide in the general Polish population used to calculate the expected number of suicides were requested from Statistics Poland.

R software (version 4.1.2) was used for statistical analysis. For numeric variables, descriptive statistics were presented as mean and standard deviation (SD). The standardized mortality ratios (SMR) were calculated for all cancers overall and for specific cancer sites based on the observed to expected number of suicide deaths. The reference was age–period–sex-specific Polish national suicide incidence rates in 5-year age groups (15–19, 20–24, …). The 95% confidence intervals (CIs) were calculated assuming a Poisson distribution. In this study, instead of presenting traditional *p* values to indicate whether the two groups differed significantly, we offer 95% CIs to define the precision of our estimate. A narrower CI indicates a more accurate estimation, whereas a wider CI is less exact. Since we compared the risk of suicide in the two groups (cancer patients and the general population) using a ratio, no difference between the two groups was indicated by a value of 1. In other words, if the ratio equals 1, the risks in these two groups are equal. Hence, if the 95% CI of the ratio contains a value of 1, the *p* value is greater than 0.05, and the difference between the groups should be considered insignificant. Alternatively, if the 95% CI does not contain a value of 1, the *p* value is strictly less than 0.05, and the difference should be considered significant.

Individual-level data from the PLCR can be used for statistics in aggregate form and for scientific purposes according to Polish legislation. The PLCR adheres to stringent regulations that ensure individual confidentiality and protection. The Strengthening the Reporting of Observational Studies in Epidemiology (STROBE) guidelines were followed [12].

## Results

From 2009 to 2019, overall, 50,298 cases (22,111 men and 28,187 women) of malignant neoplasms among AYA were registered in Poland, which supplied 147,909 person-years of observation (Table 1). Thyroid cancer (7159), testicular cancer (7117), lymphomas (6260), breast cancer (5329), and central nervous system cancer (3977) had the largest share in the total number of cases. In this study population, 38 cases of suicide were documented (31 men and 7 women).

**Table 1:** Characteristics of the study population

ICD-10	Site	Number of persons under follow-up			Person-years under risk of suicide			Number of suicides		
		Overall	Men	Women	Overall	Men	Women	Overall	Men	Women
	All cancers*	50,298	22,111	28,187	147,909	68,188	79,721	38	31	7
C00-C14 + C30	Head and neck	934	551	383	2689	1447	1242	1	1	0
C15	Esophagus	115	77	38	166	98	68	-	-	-
C16	Stomach	910	457	453	1120	533	587	1	1	0
C17	Small intestine	100	54	46	211	98	113	-	-	-
C18-C20	Colorectum	1818	946	872	3963	1982	1981	1	1	0
C21	Anus	37	20	17	69	35	34	-	-	-
C22-24	Liver and gallbladder	396	221	175	708	325	383	-	-	-
C25	Pancreas	389	187	202	647	198	449	1	1	0
C32	Larynx	64	48	16	144	86	59	-	-	-
C33	Trachea	11	4	7	29	3	26	-	-	-
C34	Lung	742	414	328	1381	785	595	1	1	0
C37	Thymus	78	39	39	206	98	108	-	-	-
C38	Heart and pleura	119	92	27	299	233	67	-	-	-
C40	Bone, limbs	694	423	271	2,283	1,311	972	-	-	-
C41	Bone, axial skeleton	500	253	247	1,388	682	707	-	-	-
C43	Melanoma	3173	1109	2064	9336	2956	6380	1	1	0
C45-C49	Soft tissues	1444	814	630	3766	1979	1787	2	2	0
C50	Breast	5329	19	5310	12,206	81	12,125	2	0	2
C51-C52	Vulva and vagina	72	-	72	138	-	138	-	-	-
C53	Cervix uteri	1821	-	1821	4458	-	4458	1	-	1
C54-C55	Corpus uteri	421	-	421	960	-	960	-	-	-
C56	Ovary	1785	-	1785	5406	-	5406	-	-	-
C60	Penis	54	54	-	95	95	-	-	-	-
C61	Prostate	24	24	-	77	77	-	-	-	-
C62	Testis	7117	7117	-	26,900	26,900	-	15	15	-
C64	Kidney	807	456	351	2046	1095	952	-	-	-
C65-C67	Bladder	510	331	179	1618	1011	607	1	1	0
C69-C72	Central nervous system	3977	2202	1775	12,313	6622	5691	3	3	0
C73	Thyroid gland	7159	1069	6090	20,918	3076	17,842	3	1	2
C74	Adrenal gland	113	51	62	279	100	179	-	-	-
C76, C80	Unspecified site	305	166	139	538	241	297	1	1	0
C81-C88	Lymphoma	6260	3119	3141	23,073	10,946	12,127	3	1	2
C90	Multiple myeloma	101	65	36	240	149	91	-	-	-
C91-C96	Leukemia	2564	1559	1005	7147	4401	2746	1	1	0

^*^All primary malignant neoplasms except non-melanoma skin cancers (C00-C43, C45-C76, and C80-C96 according to the ICD-10)

The risk of suicide for AYA after cancer diagnosis was 2.39-fold higher than that for AYA general population (SMR 2.39, 95% CI 1.69 to 3.28) (Table 2). The risk in women (SMR 4.18, 95% CI 1.68 to 8.62) was higher than that in men (SMR 2.18, 95% CI 1.48 3.09). A significantly higher risk of suicide was observed 3–5 years after diagnosis (SMR 2.58, 95% CI 1.18 to 4.90) and 5–10 years after diagnosis (SMR 3.53, 95% CI 1.76 to 6.31).

**Table 2:** Deaths due to suicide among patients with cancer standardized mortality ratios (SMR) and 95% confidence intervals (95% CI) by cancer site and sex

ICD-10	Cancer site	Overall			Men			Women		
		Observed	Expected	SMR (95% CI)	Observed	Expected	SMR (95% CI)	Observed	Expected	SMR (95% CI)
	All cancers *	38	16	2.39 (1.69–3.28)	31	14	2.18 (1.48–3.09)	7	2	4.18 (1.68–8.62)
C00-C14 + C30	Head and neck	1	0	2.91 (0.07–16.23)	1	0	3.15 (0.08–17.56)	0	0	0.00 (0.00–142.36)
C15	Esophagus	0	0	0.00 (0.00–143.19)	0	0	0.00 (0.00–152.56)	0	0	0.00 (0.00–2329.73)
C16	Stomach	1	0	7.02 (0.18–39.10)	1	0	7.77 (0.20–43.32)	0	0	0.00 (0.00–265.93)
C17	Small intestine	0	0	0.00 (0.00–151.26)	0	0	0.00 (0.00–169.44)	0	0	0.00 (0.00–1410.23)
C18-C20	Colorectum	1	1	1.98 (0.05–11.05)	1	0	2.18 (0.06–12.13)	0	0	0.00 (0.00–82.40)
C21	Anus	0	0	0.00 (0.00–413.20)	0	0	0.00 (0.00–453.39)	0	0	0.00 (0.00–4661.76)
C22-24	Liver and gallbladder	0	0	0.00 (0.00–45.76)	0	0	0.00 (0.00–51.02)	0	0	0.00 (0.00–444.35)
C25	Pancreas	1	0	17.78 (0.45–99.08)	1	0	21.33 (0.54–118.83)	0	0	0.00 (0.00–394.57)
C32	Larynx	0	0	0.00 (0.00–174.45)	0	0	0.00 (0.00–186.14)	0	0	0.00 (0.00–2776.27)
C34	Lung	1	0	4.99 (0.13–27.83)	1	0	5.37 (0.14–29.90)	0	0	0.00 (0.00–265.40)
C38	Heart and pleura	0	0	0.00 (0.00–73.95)	0	0	0.00 (0.00–76.31)	0	0	0.00 (0.00–2387.40)
C41	Bone, axial skeleton	0	0	0.00 (0.00–27.40)	0	0	0.00 (0.00–30.19)	0	0	0.00 (0.00–295.78)
C43	Melanoma	1	1	1.23 (0.03–6.83)	1	1	1.49 (0.04–8.29)	0	0	0.00 (0.00–25.73)
C45-C49	Soft tissues	2	0	4.69 (0.57–16.93)	2	0	5.09 (0.62–18.40)	0	0	0.00 (0.00–108.06)
C50	Breast	2	0	6.62 (0.80–23.91)	0	0	0.00 (0.00–193.37)	2	0	7.07 (0.86–25.52)
C51-C52	Vulva and vagina	0	0	0.00 (0.00–1172.46)	-	-	-	0	0	0.00 (0.00–1172.46)
C53	Cervix uteri	1	0	9.58 (0.24–53.36)	-	-	-	1	0	9.58 (0.24–53.36)
C54-C55	Corpus uteri	0	0	0.00 (0.00–170.80)	-	-	-	0	0	0.00 (0.00–170.80)
C56	Ovary	0	0	0.00 (0.00–31.66)	-	-	-	0	0	0.00 (0.00–31.66)
C60	Penis	0	0	0.00 (0.00–165.73)	0	0	0.00 (0.00–165.73)	-	-	-
C61	Prostate	0	0	0.00 (0.00–221.47)	0	0	0.00 (0.00–221.47)	-	-	-
C62	Testis	15	6	2.46(1.37–4.05)	15	6	2.46(1.37–4.05)	-	-	-
C64	Kidney	0	0	0.00 (0.00–14.14)	0	0	0.00 (0.00–15.35)	0	0	0.00 (0.00–180.59)
C65-C67	Bladder	1	0	4.15 (0.11–23.13)	1	0	4.39 (0.11–24.46)	0	0	0.00 (0.00–282.39)
C69-C72	Central nervous system	3	1	2.03 (0.42–5.93)	3	1	2.20 (0.45–6.43)	0	0	0.00 (0.00–32.22)
C73	Thyroid	3	1	2.87 (0.59–8.39)	1	1	1.51 (0.04–8.42)	2	0	5.21 (0.63–18.83)
C76, C80	Unspecified site	0	0	0.00 (0.00–62.23)	0	0	0.00 (0.00–68.66)	0	0	0.00 (0.00–664.44)
C81-C88	Lymphoma	3	2	1.21 (0.25–3.54)	1	2	0.45 (0.01–2.50)	2	0	8.09 (0.98–29.24)
C90	Multiple myeloma	0	0	0.00 (0.00–98.62)	0	0	0.00 (0.00–104.38)	0	0	0.00 (0.00–1787.81)
C91-C96	Leukemia	1	1	1.25 (0.03–6.96)	1	1	1.32 (0.03–7.37)	0	0	0.00 (0.00–82.70)

^*^All primary malignant neoplasms except non-melanoma skin cancers (C00-C43, C45-C76, and C80-C96 according to the ICD-10)

In a detailed risk analysis by sex and cancer site, the only significant increase in suicide risk was observed in men diagnosed with testicular cancer (SMR 2.46, 95% CI 1.37 to 4.05) (Table 2). In women, none of the cancer sites was associated with a significant increase in the risk of suicide.

Suicide risk after diagnosis of testicular cancer was further associated with time after diagnosis; the risk was significantly increased 2–3 years after diagnosis (SMR 4.23, 95% CI 1.15 to 10.83) (Table S1) and 5–10 years after diagnosis (SMR 3.82, 95% CI 1.24 to 8.92). Table S1 presents all cancers for which significant changes in suicide risk were observed when compared with the general AYA population.

## Discussion

We present the results of a large cohort study based on a national population of over fifty thousand AYA diagnosed with 34 malignancies.

The main finding of our study is that AYA has a 2.39-fold higher risk of suicide after a cancer diagnosis than the general population of 15–39-year-olds. This is an isolated observation in the European context. A large English study showed no significant differences in the risk of suicide among cancer patients aged 18–39 compared with the general population [13]. Similarly, a Lithuanian study found no significantly increased risk of suicide among patients aged 15–49 [14]. Additionally, analyses conducted in Denmark did not identify an increased risk of suicide among patients aged 0–49 years [15]. In contrast, two American studies showed a 34 times/37 times higher risk of suicide in the group of 15–39-year-olds with cancer compared to the general population [9, 16]. It is difficult to hypothesize why the Polish AYA differ from the other European AYA. Exploratory research is needed to identify the causes of the psychological burden of Polish AYA and to identify possible deficiencies in oncological care provided to this group of patients.

To the best of our knowledge, this is the second largest AYA cohort with malignancy in which suicide risk was assessed. The largest cohort came from SEER and was used in two independent AYA-concerning studies [9, 10] that presented divergent observations, making interpreting and comparing their results with ours somewhat challenging. The added value of our study in the context of the existing literature is the exceptionally granular assessment of suicide risk in the AYA population after cancer diagnosis. This is the first study to quantify the risk of suicide in 34 malignancies in this age group, and the first study to stratify this risk over time from diagnosis.

In detailed analyses, we identified men diagnosed with testicular cancer as a suicide risk group, especially in 2–3 and 5–10 years after diagnosis. No previous literature has described the risk of suicide after a diagnosis of testicular cancer among AYA (independent of how it was defined). However, in studies concerning the entire population of cancer patients, regardless of age, the results differed by country. In England and Norway, no significant differences were found between suicide rates among patients with testicular cancer and the general population [13, 17]. However, such differences were found in the USA, where the SMR of suicide after the diagnosis of testicular cancer in the period < 1 year from the diagnosis was 6.31 (95% CI 1.30 to 18.43), after 1–5 years SMR was 11.63 (95% CI 7.59 to 17.03), and > 5 years SMR was 17.66 (95% CI 13.94 to 22.07) [16].

Men diagnosed with testicular cancer are significantly more likely to experience anxiety and depression [18, 19]. In a detailed study on the risk of suicide after a diagnosis of testicular cancer based on SEER data, only age < 30 years was associated with an increased suicide risk [18]. Race, marital status, stage, and decade of cancer diagnosis were unrelated [18]. It is difficult to determine the reasons for the increased risk of suicide in this group of patients unequivocally. However, the fact that both our analysis and the American study [16] showed an increase in risk over time after diagnosis allows for diverse hypotheses. Physical-, mental-, and job-related problems, as well as shifting outlooks on life and at work, may affect personal and professional judgments among long-term survivors of testicular cancer [20], which, in the broader perspective, may be linked to increased suicide risk.

The main advantages of the present study include coverage of the entire national population (representability and generalizability), large study population (over fifty thousand individuals), and calculation model (age–sex–year-SMR). Even so, the study did not have enough power to identify groups of women at an increased risk of suicide at particular cancer sites. In addition, although statistically significant, some of the results were based on individual events, and in such cases, the chance findings cannot be ruled out. Therefore, although we present these results in the supplementary materials, we do not draw any conclusions based on them. We do not propose that they should become the basis for making decisions about changes in clinical practice regarding the care of AYA with cancer. An extensive international study of a larger population could provide more information about particular risk groups. In addition, a limitation may be that, as in other studies based on large cohorts sourced from national registries, we had no access to history of substance use and pre-existing mental health diagnoses, which are examples of potential confounding factors.

## Conclusions

After cancer diagnosis, Polish individuals aged 15–39 years are almost 2.5 times higher risk of suicide than the general AYA population. Men with testicular cancer within 2–3 and 5–10 years after cancer diagnosis constitute a particular risk group. It is necessary to create easy-to-use tools to detect more vulnerable cancer patients or to adapt existing tools that screen for pre-existing or new mental health conditions such as depression or anxiety, which could be employed during oncological follow-up. Other measures that should be undertaken include educating cancer care providers and family physicians and integrating psychological services into select cancer care specialties.

## Supplementary Information

Below is the link to the electronic supplementary material.

Supplementary file1 (DOCX 26 KB)

## Data Availability

The data analyzed in this study were obtained from the PLCR and are available upon reasonable request by contacting the PLCR at krn@pib-nio.pl and subject to ethical approvals in place and material transfer agreements.
