# Supplementary material for: Suicide risk among adolescents and young adults after cancer diagnosis: analysis of 34 cancer groups from 2009 to 2019
Source: J Cancer Surviv. 2023 Mar 17;17(3):657–62. doi: 10.1007/s11764-023-01358-5 (PMC10209251; doi:10.1007/s11764-023-01358-5)
Supplement: Supplementary file 1 — Supplementary file1 (DOCX 26 KB) [file 11764_2023_1358_MOESM1_ESM.docx]

**Suicide risk among adolescents and young adults after cancer diagnosis: analysis of 34 cancer groups from 2009 to 2019.** Irmina Maria Michalek^1^; Florentino Luciano Caetano dos Santos^1^; Urszula Wojciechowska^1^; Joanna Didkowska^1^. **Journal of Cancer Survivorship**

Affiliations: 1 – Polish National Cancer Registry, Maria Sklodowska-Curie National Research Institute of Oncology, Warsaw, Poland

Corresponding author:

Irmina Maria Michalek, MD, PhD

Polish National Cancer Registry

Maria Sklodowska-Curie National Research Institute of Oncology

ul. Wawelska 15B, 02-093 Warsaw, Poland

e-mail: [irmina.michalek@pib-nio.pl](mailto:irmina.michalek@pib-nio.pl)

**Table S1.** Deaths due to suicide among male patients with cancer – standardized mortality ratios (SMR) and 95% confidence intervals (95% CI) by cancer site and time since diagnosis.

| **ICD-10** | **Site** | **Time since diagnosis [years]** | **Observed** | **Expected** | **SMR (95% CI)** |
| --- | --- | --- | --- | --- | --- |
|  | **All cancers*** | [0.0, 0.5) | 5 | 2.08 | 2.40 (0.78-5.61) |
|  |  | [0.5, 1.0) | 2 | 1.82 | 1.10 (0.13-3.97) |
|  |  | [1.0, 2.0) | 4 | 3.02 | 1.33 (0.36-3.40) |
|  |  | [2.0, 3.0) | 6 | 2.41 | 2.49 (0.91-5.43) |
|  |  | [3.0, 5.0) | 9 | 3.48 | 2.58 (1.18-4.90) |
|  |  | [5.0,10.0] | 11 | 3.12 | 3.53 (1.76-6.31) |
| **C16** | **Stomach** | [0.0, 0.5) | 0 | 0.05 | 0.00 (0.00-79.13) |
|  |  | [0.5, 1.0) | 0 | 0.03 | 0.00 (0.00-128.58) |
|  |  | [1.0, 2.0) | 0 | 0.03 | 0.00 (0.00-133.84) |
|  |  | [2.0, 3.0) | 0 | 0.01 | 0.00 (0.00-280.61) |
|  |  | [3.0, 5.0) | 0 | 0.01 | 0.00 (0.00-252.27) |
|  |  | [5.0,10.0] | 1 | 0.01 | 84.32 (2.13-469.79) |
| **C25** | **Pancreas** | [0.0, 0.5) | 0 | 0.02 | 0.00 (0.00-205.51) |
|  |  | [0.5, 1.0) | 0 | 0.01 | 0.00 (0.00-359.02) |
|  |  | [1.0, 2.0) | 0 | 0.01 | 0.00 (0.00-348.11) |
|  |  | [2.0, 3.0) | 0 | 0.01 | 0.00 (0.00-596.57) |
|  |  | [3.0, 5.0) | 0 | 0.01 | 0.00 (0.00-590.00) |
|  |  | [5.0,10.0] | 1 | 0 | 200.88 (5.09-1119.22) |
| **C53** | **Cervix uteri** | [0.0, 0.5) | 1 | 0.02 | 55.49 (1.40-309.19) |
|  |  | [0.5, 1.0) | 0 | 0.02 | 0.00 (0.00-239.59) |
|  |  | [1.0, 2.0) | 0 | 0.02 | 0.00 (0.00-162.48) |
|  |  | [2.0, 3.0) | 0 | 0.02 | 0.00 (0.00-235.36) |
|  |  | [3.0, 5.0) | 0 | 0.02 | 0.00 (0.00-190.60) |
|  |  | [5.0,10.0] | 0 | 0.01 | 0.00 (0.00-277.91) |
| **C62** | **Testis** | [0.0, 0.5) | 2 | 0.68 | 2.96 (0.36-10.70) |
|  |  | [0.5, 1.0) | 1 | 0.63 | 1.59 (0.04-8.88) |
|  |  | [1.0, 2.0) | 1 | 1.12 | 0.89 (0.02-4.98) |
|  |  | [2.0, 3.0) | 4 | 0.95 | 4.23 (1.15-10.83) |
|  |  | [3.0, 5.0) | 2 | 1.43 | 1.40 (0.17-5.05) |
|  |  | [5.0,10.0] | 5 | 1.31 | 3.82 (1.24-8.92) |
| **C73** | **Thyroid** | [0.0, 0.5) | 0 | 0.14 | 0.00 (0.00-26.85) |
|  |  | [0.5, 1.0) | 0 | 0.12 | 0.00 (0.00-29.86) |
|  |  | [1.0, 2.0) | 0 | 0.21 | 0.00 (0.00-17.31) |
|  |  | [2.0, 3.0) | 0 | 0.17 | 0.00 (0.00-21.88) |
|  |  | [3.0, 5.0) | 3 | 0.22 | 13.57 (2.80-39.67) |
|  |  | [5.0,10.0] | 0 | 0.18 | 0.00 (0.00-20.35) |

***** All primary malignant neoplasms except non-melanoma skin cancers (C00-C43, C45-C76, C80-C96 according to the ICD-10)
